# Aging-related inflammatory and metabolic disorder in the novel mutation of *colony-stimulating factor-1 receptor (csf1r)*^*P853T/+*^ in CSF1R-microglial encephalopathy

**DOI:** 10.1016/j.gendis.2024.101289

**Published:** 2024-04-05

**Authors:** Xiaohong Wang, Yanli Wang, Tianlin Jiang, Jiwei Jiang, Linlin Wang, Shiyi Yang, Mengfan Sun, Yuan Zhang, Ziyan Jia, Wenyi Li, Qiwei Ren, Cuicui Zhang, Jianjian Liu, Yinwei Zhu, Min Zhao, Shirui Jiang, Huiying Zhang, Jinglong Chen, Jun Xu

**Affiliations:** a Institute of Translational Medicine, Medical College, Yangzhou University, Yangzhou, Jiangsu 225000, China; b Department of Neurology, Beijing Tiantan Hospital, Capital Medical University, Beijing 100070, China; c School of Public Health, Shandong Second Medical University, Weifang, Shandong 261000, China; d Department of Neurology, The Second Affiliated Hospital of Xuzhou Medical University, Xuzhou, Jiangsu 221000, China; e Department of Neurology, Fuxing Hospital, Capital Medical University, Beijing 100045, China; f Department of Neurology, Changshu Hospital Affiliated to Soochow University/Changshu First People's Hospital, Changshu, Jiangsu 215500, China; g Department of Geriatric Medicine, Guangzhou First People's Hospital, School of Medicine, South China University of Technology, Guangzhou, China

CSF1R-microglial encephalopathy (CME) is a rare dementia with rapid development of cognitive impairment. The main clinical features of CME are progressive cognitive impairment, motor dysfunction, and neuropsychiatric symptoms.^1^ Pathological features of CME showed diffuse leukoencephalopathy and thin corpus callosum, and immunohistochemical staining showed axonal bulbous transformation with pigmentary glial cells, extensive axonal degeneration, and myelin loss. The clinical phenotypes of CME are complex and varied, including progressive cognitive decline, movement disorders, seizures, psychobehavioral abnormalities, and multiple complications. The initial clinical manifestations of the disease vary greatly and are non-specific, so it is easy to be missed or misdiagnosed as Alzheimer's disease, multiple sclerosis, or other leukoencephalopathy. The average age of onset is 8–72 years (mean: 42 years) and the prognosis is poor with a median survival of 2–30 years (mean: 6 years). Current treatments include microglia replacement and symptomatic treatments, with no specific treatments for now. The main reason for this is that CME is difficult to diagnose because of its unknown mechanism, often requiring highly expensive gene sequencing.^2^ Most CSF1R mutation models reported to date are all knockout models, which cannot well mimic the clinical phenotype.^3^ CSF1R haploinsufficiency model mice show olfactory dysfunction, myelin hyperplasia, and increased reverse transcriptase of cortical oligodendrocytes, which have not been described in clinical lines.^4^ There has been no effective model of CME, and extensive knockout will cause gene deletions resulting in lethal defects. In this article, we proved that the *CSF1R*^*P853T/+*^ model can well imitate CME. This article explores the mechanisms of CME in the *CSF1R*^*P853T/+*^ mouse model and finds that CME suffers from abnormal intercellular communication, mitochondrial malformations, and enlarged lysosomes, worsening inflammation in the brain, which in turn exacerbate mitochondrial damage and form a vicious circle of predominantly immuno-inflammatory senescence. All animal protocols were approved by Yangzhou University's Institutional Animal Care and Use Committee and Animal Ethics Committee (No. YXYLL-2022-71), China, in accordance with the NIH Guidelines for the Care and Use of Laboratory Animals.

Behavioral assessments of this model are consistent with clinical findings. Among all ages of *Csf1r*^*P853T/+*^ mice, 10-month-old mice behaved the most typical features, in the Morris water maze (Fig. S2A), beam test (Fig. S2B), and Luxol fast blue staining (Fig. S2C). Morris water maze showed that *Csf1r*^*P853T/+*^ mice display significant learning and cognitive deficits (Fig. 1A). The time to identify the platform was significantly increased and the number of times mice entered the platform was reduced in *Csf1r*^*P853T/+*^ mice compared with those in *Csf1r*^*flox*^ (wild-type/WT) mice. The results of the beam test are presented in Figure 1B, the duration and error times crossing the beam were much higher in *Csf1r*^*P853T/+*^ mice than those of WT mice. These results suggest that *Csf1r*^*P853T/+*^ mice exhibit significant motor impairment. To assess the depression-like behavior, forced swimming test, tail suspension test, and sucrose preference test were conducted (Fig. S1A–C). The forced swimming test revealed that *Csf1r*^*P853T/+*^ mice had significantly longer immobility time than WT mice, indicating that their depressive symptoms were induced by *Csf1r*^*P853T/+*^ mutation (Fig. S1A). The tail suspension test indicated that the immobility duration of *Csf1r*^*P853T/+*^ mice was longer than that of WT mice (Fig. S1B). In the sucrose preference test, and the depressive symptoms of *Csf1r*^*P853T/+*^ mice were further intensified (Fig. S1C). The results of the prepulse inhibition experiment are presented in Figure S1G. The prepulse inhibition rate of *Csf1r*^*P853T/+*^ mice under prepulse stimulation was significantly lower than that of WT mice. The results of the attack behavior test are presented in Figure S1D–F. The attack latency of *Csf1r*^*P853T/+*^ mice was significantly lower than that of WT mice, while the attack times of *Csf1r*^*P853T/+*^ mice were much higher. These findings suggest that *Csf1r*^*P853T/+*^ mice display schizophrenia-like behavioral deficits.

**Fig. 1:**
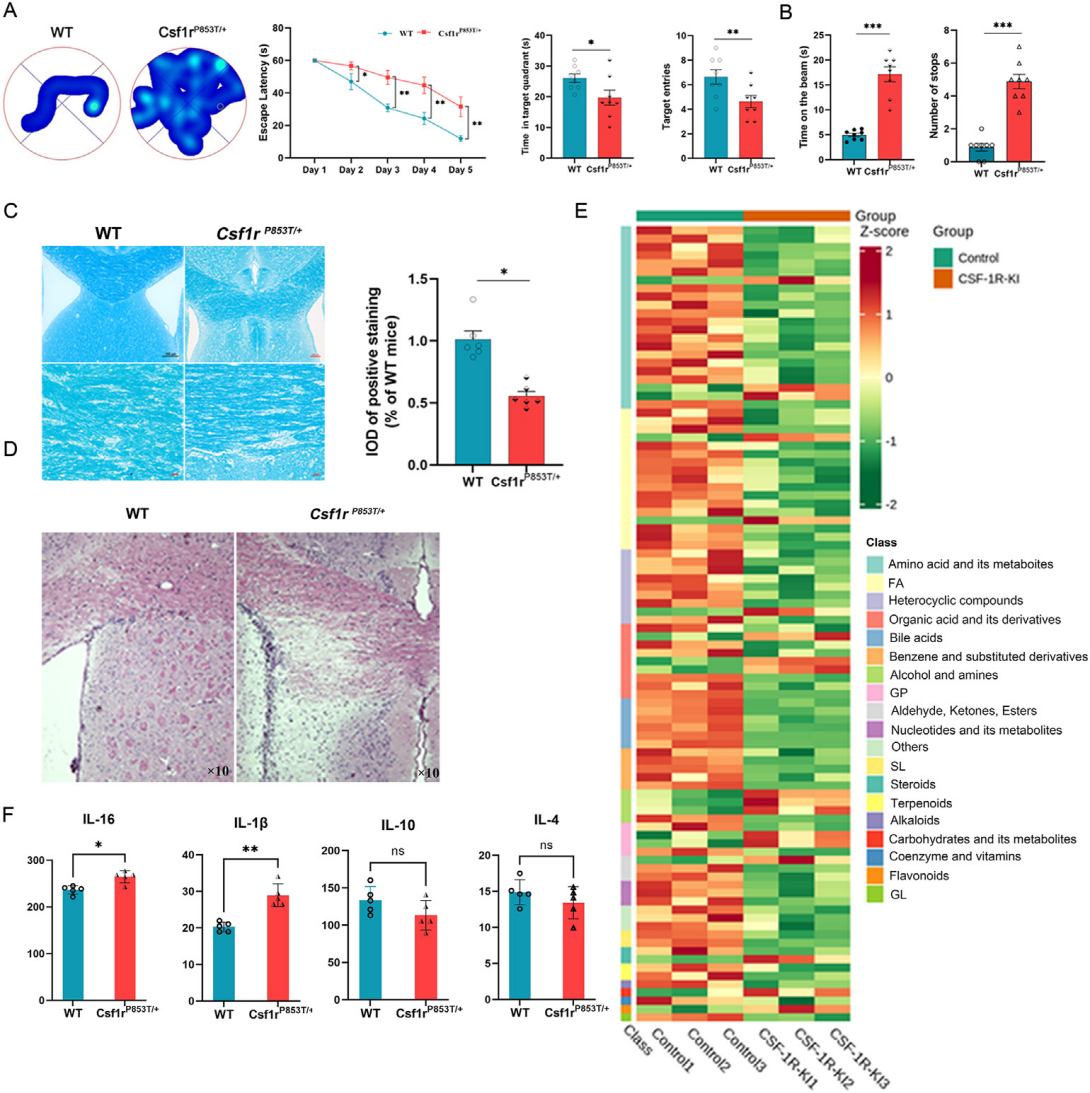
*Csf1r*^*P853T/+*^ mice develop cognitive impairment and motor dysfunction via the myelination process, mitochondrial dysfunction, and synaptic loss in comprehensive pathways. **(A)** Morris water maze test for assessment of memory function. **(B)** Beam test for assessment of motor function. **(C)** Luxol fast blue staining for the corpus callosum. **(D)** Hematoxylin and eosin staining for the corpus callosum. **(E)** The metabolite set enrichment analysis map indicated the metabolic enrichment analysis of the most relevant metabolic pathway related to *Csf1r*^*P853T/+*^ mutation in mice. The data were shown as mean ± standard error of the mean. ∗*P* < 0.05, ∗∗∗*P* < 0.001. CSF-1R–KI stands for *Csf1r*^*P853T/+*^.

In *Csf1r*^*P853T/+*^ mice, hematoxylin and eosin staining and Luxol fast blue staining both indicate thinning corpus callosum, uneven fiber arrangement, and vacuolation (Fig. 1C, D). This suggests consistency with clinical imaging suggestive of deep white matter degeneration. Electron microscopy results showed enlarged lysosomes in the frontal lobe, ruptured mitochondria in the hippocampus, and abnormal morphology of myelin sheaths in the corpus callosum suggesting altered intercellular communication (Fig. S3A). Immunofluorescence staining shows that higher levels of Iba-1 and alpha-B in the deep brain cortex of *Csf1r*^*P853T/+*^ mice suggest microglia- and macrophage-specific accumulation (Fig. S3C).

In conclusion, the behavioral and pathology features in *Csf1r*^*P853T/+*^ mice are consistent with CME clinical features. To further investigate the mechanism, inflammatory cytokines were tested. Results showed that IL-16 and IL-1β levels in *Csf1r*^*P853T/+*^ mice were higher compared with WT mice, while IL-4 and IL-10 showed no significance (Fig. S3B). This indicates the activation of microglia, releasing proinflammatory cytokines and exacerbating the inflammatory response in the brain.

Gene set enrichment analysis showed that the most enrichment pathway was the structural constituent of the myelin sheath (Fig. S4A). Among all the significant pathways, pathways associated with myelin and glial cells accounted for the largest proportion, which is also consistent with pathological observations.

Subsequently, we analyzed the overall metabolic levels of *Csf1r*^*P853T/+*^ mice. Heat maps of metabolic analysis suggested that amino acid and fatty acid metabolism differed significantly in *Csf1r*^*P853T/+*^ mice (Fig. S4B). Metabolite set enrichment analysis represented the most relevant metabolic pathways in *Csf1r*^*P853T/+*^ mice, and the top three among them were steroid synthesis, fatty acid degradation, and primary bile acid synthesis. Amino acid and fatty acid metabolism, as major energy metabolism pathways, can significantly affect energy synthesis in *Csf1r*^*P853T/+*^ mice, resulting in aging (Fig. 1E).

In this article, the behavioral, pathological, inflammatory cytokine levels, gene enrichment analysis, and metabolic analysis confirmed that *Csf1r*^*P853T/+*^ mice can compensate for the shortcomings of previous models, and preliminary mechanism studies were conducted experimentally, which were consistent with aging phenotype dominated by abnormal energy metabolism and immunoinflammation, providing a basic for subsequent mechanism studies and drug development (Fig. S5).

## Author contributions

Conceptualization, Xu J, Wang XH; methodology, Wang YL and Jiang TL; validation, Wang XH and Wang YL; formal analysis, Jiang JW; investigation, Wang LL; resources, Yang SY, Sun MF, Zhang Y, Jia ZY, Li WY; data curation, Wang XH; writing-original draft preparation, Wang YL, Jiang TL; writing-review & editing, Xu J, Jiang JW; visualization, Ren QW, Zhang CC, Liu JJ, Zhu YW; supervision, Xu J; project administration, Xu J; funding acquisition, Xu J.

## Conflict of interests

The authors declare no conflict of interests.

## Funding

This study was supported by the 10.13039/501100001809National Natural Science Foundation of China (No. 82071187, 81870821, 81501135, and 81471215), the 10.13039/501100012166National Key Research and Development Program of China (No. 2021YFC2500100, 2021YFC2500103), the Beijing Youth Talent Team Support Program (China) (No. 2018000021223TD08), and Science and Technology Program of Guangzhou (China) (No. 202201020343).

## Figures and Tables

**figs1:**
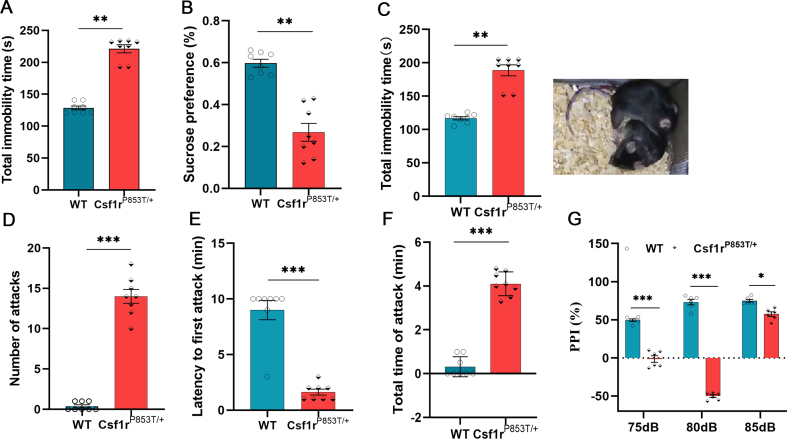


**figs2:**
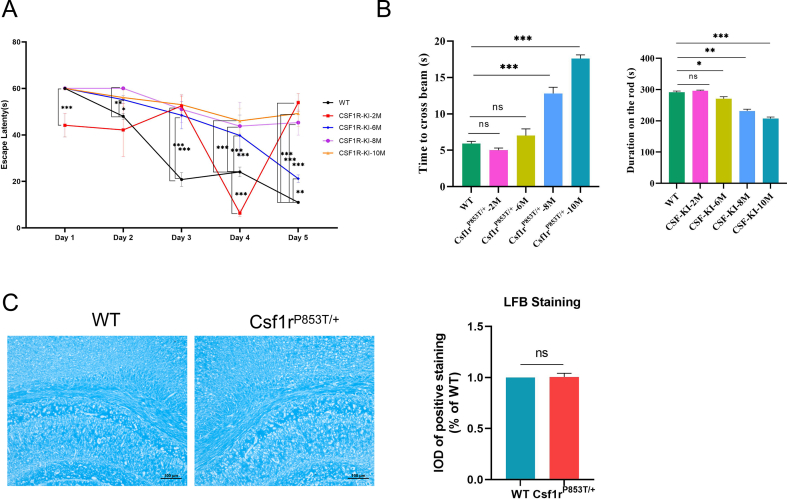


**figs3:**
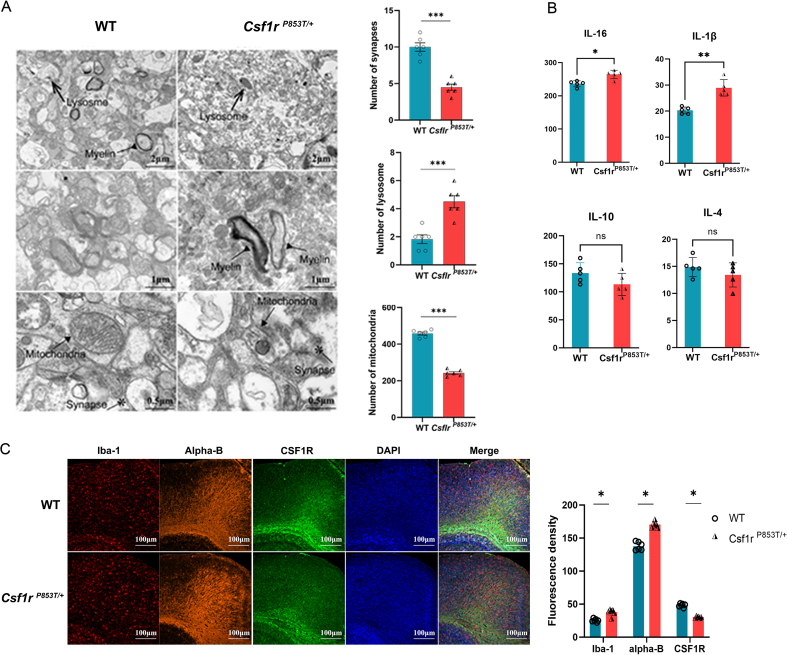


**figs4:**
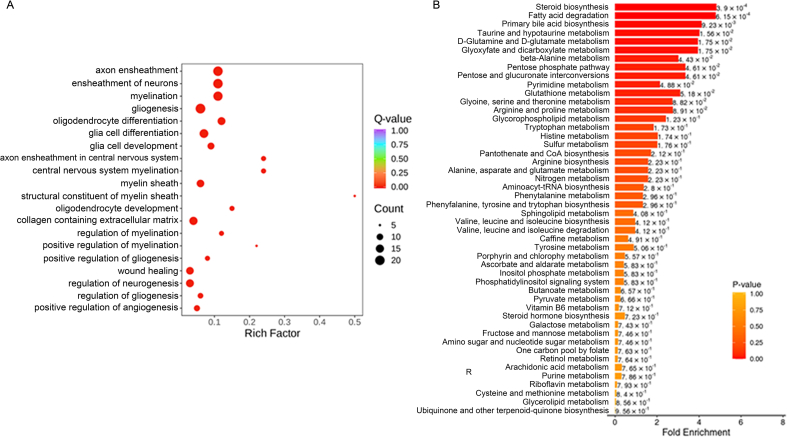


**figs5:**
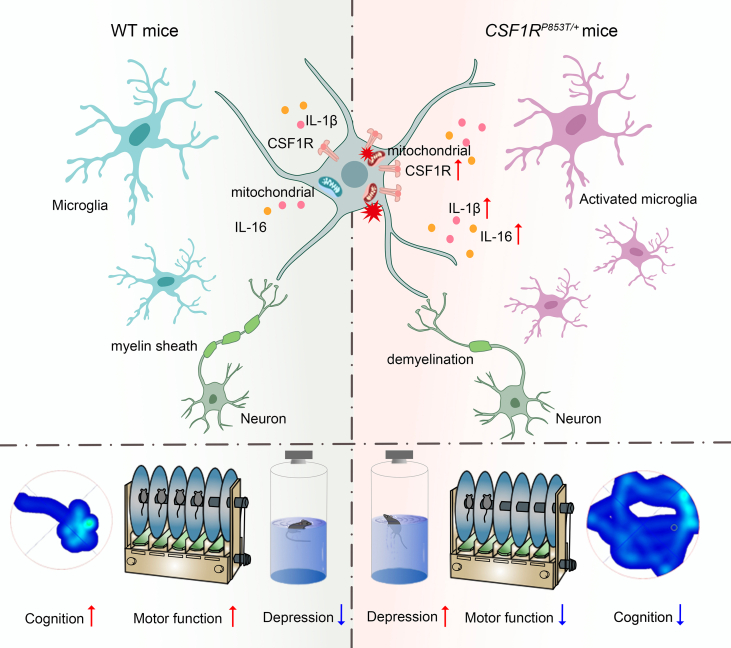


## References

[1] Konno T., Yoshida K., Mizuta I. (2018). Diagnostic criteria for adult-onset leukoencephalopathy with axonal spheroids and pigmented glia due to CSF1R mutation. Eur J Neurol.

[2] Papapetropoulos S., Pontius A., Finger E. (2022). Adult-onset leukoencephalopathy with axonal spheroids and pigmented *Glia*: review of clinical manifestations as foundations for therapeutic development. Front Neurol.

[3] Oosterhof N., Kuil L.E., van der Linde H.C. (2018). Colony-stimulating factor 1 receptor (CSF1R) regulates microglia density and distribution, but not microglia differentiation *in vivo*. Cell Rep.

[4] Li X., Hu B., Guan X. (2023). Minocycline protects against microgliopathy in a Csf1r haplo-insufficient mouse model of adult-onset leukoencephalopathy with axonal spheroids and pigmented glia (ALSP). J Neuroinflammation.

