# Supplementary material for: Aging-related inflammatory and metabolic disorder in the novel mutation of colony-stimulating factor-1 receptor (csf1r)P853T/+ in CSF1R-microglial encephalopathy
Source: Genes Dis. 2024 Apr 5;12(2):101289. doi: 10.1016/j.gendis.2024.101289 (PMC11635720; doi:10.1016/j.gendis.2024.101289)
Supplement: Multimedia component 1 [file mmc1.docx]

**Supplementary materials and methods**

**Animals**

The generation, maintenance, and genotyping of *Csf1r* knock-in (KI) mice were conducted in collaboration with Cyagen. *Csf1r ^P853T/+^* mice were generated by crossing *Csf1r*^flox^ mice with *Csf1r*^KI^ mice. Littermate wide type mice were used as controls. The animals were housed under an air conditioned (22±1 ℃) and 12-h light/dark cycle-regulated room with standard lab chow and water available ad libium. All the animals were taken care of according to international standard for 1 week prior to experiment to make them amenable.

The behavioral studies (10 months of age) involved 10 male *Csf1r ^P853T/+^* mice and 10 age matched wide type mice. Histopathologic analysis was carried out at 10 months of age utilizing a subgroup of 10 male mice and their controls. Additional *Csf1r ^P853T/+^* and wide type mice were subject to ultrastructural studies at 10-months of age.

**Behavioral test**

**Morris water maze**

Every experiment was carried out in a setting appropriate for behavioral assessment. The experimental setup for the water labyrinth involved a circular container with a diameter of 120 cm, filled with water at a temperature range of 20-22°C. Additionally, an exit platform made of PVC with a diameter of 10 cm was positioned 0.8 cm below the water surface. The spatial trial sessions consisted of four rounds each, with randomized starting positions. Prior to being instructed to remain on the platform for a duration of 15 seconds, the mice were granted a period of 60 seconds to engage in exploratory behavior on said platform. In the event that the rodent failed to locate the platform within a time frame of 60 seconds, it was subsequently directed towards the platform and remained in situ for a duration of 15 seconds. On the sixth day, the investigative examination was carried out, wherein the concealed platform of the pool was eliminated. During the experimentation, the rodents were allotted a duration of 60 seconds to investigate the pool. The study utilized a computerized video tracking system (VisuTrack 2.0, Xinruan, Co., Shanghai, China) to collect behavioral data on animals.

**Beam test**

The beam test is a method employed to evaluate the motor coordination and balance of mice. A balance beam, about 1 meter in length, was arranged for the mice to traverse. The mice were positioned at the starting point, and a powerful light beam was employed to motivate them to traverse the beam to reach the opposite end. The rodents underwent a 3-day training period to traverse the length of the beam, culminating in a 30-second period of rest within the dark enclosure located at the end of the beam. The mice underwent a training regimen of three sessions per day until they achieved the ability to traverse the beam unassisted and without interruption. On the fourth day of the experiment, the test was initiated and the duration required to traverse the entire length of the balance beam, starting from its origin, was documented. A shorter duration of time results in an improved balance of the mice. In order to mitigate the impact of individual variability in behavioral experiments, the balance beam was utilized to test three cohorts of mice.

**Forced swimming test**

In order to assess behavioral discomfort in the Forced Swimming Test (FST), the test subjects, i.e. mice, were permitted to swim in a 2-liter glass container filled with water up to 75% of its volume. Prior to each experiment, the beaker underwent a thorough cleaning process. The temperature of the water was maintained within the range of 21 to 25℃. A total of six minutes of their behavior were recorded on video, with the final four minutes being subjected to analysis. All possible measures were taken to minimize the impact of light that was reflected from the water's surface. The light intensity of 100-120 lx was situated at the beaker level, which was 10 feet above the behavioral setup. To reduce glare, a matte-finished, non-reflective white covering was positioned underneath the beakers. The camera was positioned in a horizontal orientation at a distance of three feet from the beakers, and was situated at the same level as the water. The camera and beakers remained stationary throughout several days of recorded footage. When the mouse remained motionless and made only minimal movements to maintain its head above water, it was deemed immobile for manual analysis. In comparison to the total duration, the duration of sedentary behavior was recorded in seconds (100s).

**Prepulse inhibition analysis**

The prepulse inhibition (PPI) experiment was carried out using a Startle Reflex (Med Associates Inc, USA). Prior to the commencement of the experiment, a period of 5 minutes was allocated for the acclimatization of the mice within the experimental apparatus.

The ambient noise level was measured at 68 decibels across 86 experimental trials. The study employed eight distinct trial types, including the pulse-alone stimulus at 120 dB for 40 ms, three prepulse-alone stimuli at 3, 6, and 12 dB above the background sound for 20 ms, three combined prepulse-alone stimuli (pulse+prepulse), and three prepulse-alone stimuli for 20 ms (pulse+prepulse, with a 100 ms interval between them). Additionally, a trial with no stimulus was included, where only the background sound was presented. The researcher presented the eight types of trials in a pseudo-random manner, with each type being presented 10 times and an average inter-trial interval of 15 seconds (ranging from 8 to 22 seconds). Furthermore, the experimental protocol involved administering 6 distinct startle reflex stimuli at the outset of the test, with the intention of minimizing the initial response of the animal to a steady state level. It is noteworthy that the outcomes of these stimuli were not incorporated in the subsequent analysis. The present study aims to assess the performance of the sensory gating system in mice.

**Attack test**

We used the fear conditioning paradigm (XRXC404, Softmaze Information Technology Co. Ltd., Shanghai, China; 30 cm in length, 26 cm broad, 22 cm high) to assess hippocampus-related learning and memory. On the day of the acquisition, mice were familiarized for 2 min, given four 2-s, 0.4 mA foot shocks spaced 2 min apart, and then put back in cages for a further 2 min. The day 2 experiment was conducted the following day in the identical chamber for 6 minutes by assessing the freezing time as a measure of fear memory.

**Sucrose preference test**

The measurements conducted on the sixth day were taken during the nighttime period, specifically between the hours of 7 a.m. and 10 p.m. The daytime measurements encompassed the period from 6 p.m. on the initial day to 9 a.m. on the seventh day. Ensure that the apparatus has been adequately set up as instructed during this duration. Activate the electrical power supply for the SPT electronic apparatus on the second day. Following the deprivation period at 9:00 a.m., transfer the home cages to the designated testing area and employ a randomized approach to allocate mice from their respective home cages to the apparatus chambers. To commence data collection for the experiment, please click on the "start" button. Upon reaching a predetermined detection length, the software-MDA will initiate an automatic pause. Reinstate the mice to their initial enclosures and afford them unimpeded availability of sustenance and hydration. To conduct an analysis of the data, the user is advised to select the "analysis" option on the MDA software. The software allows for the selection of a period of either 30 or 60 minutes, or a custom period. The trial results can then be exported for further analysis. The data obtained includes the consumption periods and total time for both sucrose water and plain water. Utilize the following equations to ascertain the degree of preference for sucrose: The preference of an individual for sucrose can be quantified by calculating the ratio of the duration of sucrose consumption to the total duration of consumption, and then multiplying this ratio by 100%. The aggregate duration of sugar and regular water consumption is equivalent to the total consumption time. Thoroughly sanitize the laboratory. Ultimately, employ a solution consisting of 70% ethanol to eliminate any lingering scents.

**Tail suspension test**

During the Tail Suspension Test (TST), mice were subjected to a six-minute suspension by their tails. As previously stated, it has been observed that C57/BL6 mice display a notable tendency towards tail climbing. In order to address this behaviour, a lightweight plastic tube weighing 0.5 g was utilised to passively restrict tail movement and prevent tail climbing. The camera placement and illumination configurations were analogous to those employed in FST. Following the experimental trials, the mice were transferred to a containment enclosure and held therein until all the mice residing in their communal cage had undergone testing. The period of motionlessness was documented and juxtaposed with the overall duration of 240 seconds.

**HE and LFB staining**

After sectioning the wax block of mouse brain tissue, paraffin sections were dewaxed, entered into water, patched and stained with Nisin stain; washed and divided in water, divided in 95% ethanol for 5 min until the cell granules were clearly visible; dehydrated and sealed with neutral gum; finally microscopic examination, photographs were taken to observe the morphology of brain.

The tissue sections underwent a washing process using distilled water, followed by immersion in a 0.1% LFB solution and subsequent sealing at 60°C for a duration of 8-16 hours. Following the washing process with distilled water, the sections were immersed in 95% alcohol. The sections were subjected to partitioning using an aqueous solution of lithium carbonate with a concentration of 0.05% for a duration exceeding 10 seconds. The process of colour separation was extended through the use of 70% alcohol until the differentiation between the grey and white matter was distinctly discernible under microscopic examination. The sections underwent a washing process utilising distilled water, followed by a restaining procedure utilising a 0.25% tar violet solution supplemented with a small quantity of glacial acetic acid for a duration of 10 minutes. A solution consisting of 70% alcohol was employed to extract the residual dye until the nuclei and nictitating membranes attained a red hue. The film underwent a drying process on filter paper, followed by two rinses in n-butanol and subsequent dehydration for a duration of 3-5 minutes per cycle. The film was cleared using xylene and subsequently sealed with neutral gum. The myelin sheath exhibits a vivid blue hue, while the nucleus appears as a deep shade of blue.

**Immunofluorence and immunostaining analysis**

Three animals were perfused intracardically with 25ml sterile saline and 50ml 4% paraformaldehyde (PFA) in 0.1M PB, mice brains were rapidly removed and post-fixed in 4% PFA for 48 hr at 4℃, then were cryoprotected in 15%, 20% and 30% sucrose for 24hr sequentially till the brains sunk. The brains were frozen in isopentane on the liquid nitrogen and sectioned serially into 8 μm-thick sections through the midbrain. For the immunofuorescence staining, brain sections were washed with PBS and were blocked with 5% normal goat serum for 0.5 h at room temperature, and then incubated were incubated with rabbit polyclonal anti-CSF-1R antibody (ab254357, Abcam plc, Cambridge, UK), anti-Alpha B Crystallin (ab281561, Abcam plc, Cambridge, UK), and anti-Iba-1 (E4O4W, Cell Signaling Technology, Inc, USA) overnight at 4 ℃. Then slices were incubated with the Fluorescein (FITC)-conjugated affinipure goat anti-mouse IgG (115-095-003, Jackson ImmunoResearch), CY^TM3^-conjugated affinipure goat anti-rabbit (111-165-003, Jackson ImmunoResearch, USA). The slices were incubated with DAPI for 5 min. The expression of CSF1R, Alpha B, and Iba-1 in neurons was collected under a fuorescence microscope using Image-Pro Plus 6.0 (Media Cybernetics, Silver Spring, MD, USA).

**Electron microscope scanning**

The fixed prefrontal lobe, corpus callosum, hippocampus tissues were dehydrated, soaked, embedded, baked at 45 °C for 12 h and then transferred to a 72 °C oven for 24 h. The tissues were sectioned (70 nm), retrieved, electronically stained, and photographed by transmission electron microscopy. Five fields of view were selected for filming.

**Luminex Test**

Prepare the standard, bead premix, detection antibody, PE-streptavidin and washing solution according to the manufacturer's instructions. Add 25 μL of standards, samples and magnetic beads into the wells of 96-well microchip, seal the membrane, and incubate the microchip overnight at 800 r/min under a flat plate shaker to protect the light, then wash the microchip for 3 times, add 25 μL of detection antibody into the wells, seal the membrane, and incubate the microchip at 800 r/min under a flat plate shaker to protect the light for 1 h. Wash the microchip again, then add 25 μL of PE-Streptavidin into the wells, seal the membrane, and incubate it for 0.5 h at 800 r/min. After washing the chip again, add 25 μL of PE-Streptavidin into the wells, seal the membrane, and incubate at 800 r/min on a plate shaker for 0.5 h. Wash the chip for 3 times, add 100 μL of sheath solution, seal the membrane, resuspend the beads, and incubate at 800 r/min for 2 min on a plate shaker, and then send it to the calibrated Luminex 200^TM^ instrument for detection to get the final results of the cytokine concentration.

**RNA extraction and Illumina NovaSeq Sequencing**

The extraction of total RNA was performed by grinding 10 mg of tissues using a Homogenizer (Scientz) and utilizing TRIzol® Reagent (Invitrogen) and RNeasy minElute spin column (Qiagen) in accordance with the manufacturer's guidelines at Mingma Technologies Co., Ltd. located in Shanghai. Subsequently, the quality of the total RNA (Thermo Scientific) was assessed using the 2100 Bioanalyser (Agilent) and the NanoDrop. In order to generate a sequencing library, a quantity of approximately 500 nanograms of RNA sample that exhibited high quality characteristics (specifically, an OD260/280 ratio between 1.9 and 2.0, and a RIN value greater than or equal to 8) was utilized.

Following the construction of the library, the quantification of sequencing libraries was performed using a Qubit 3.0 fluorometer dsDNA HS Assay (Thermo Fisher Scientific), and the size distribution was assessed using an Agilent BioAnalyzer (Agilent). The sequencing process was conducted at Mingma Technologies Co., Ltd. located in Shanghai, utilizing an Illumina system and following the prescribed protocols provided by Illumina for 2x150 paired-end sequencing.

**Untargeted metabolomics analysis**

The ProteoWizard software was utilized to convert the LC-MS acquired data file into the mzML format. The XCMS program was utilized to execute peak extraction, peak alignment, and retention time correction procedures. The peak area was corrected using the "SVR" method. Samples with peak detection rates below 50% were excluded from each group. Subsequently, metabolic identification data was acquired through a comprehensive search of the laboratory's proprietary database, publicly available databases, an artificial intelligence database, and metDNA.

The unsupervised application of principal component analysis (PCA) was executed through the utilization of the prcomp statistical function within the R programming language (available at www.r-project.org). Prior to unsupervised PCA, the data underwent unit variance scaling.

The findings of the study involved the utilization of hierarchical cluster analysis (HCA) to analyze the samples and metabolites, which were then visually represented as heatmaps accompanied by dendrograms. Additionally, the Pearson correlation coefficients (PCC) between the samples were computed using the cor function in R and were solely presented as heatmaps. The ComplexHeatmap R package was utilized to perform both HCA and PCC.

The metabolites that were identified underwent annotation through utilization of the KEGG Compound database (http://www.kegg.jp/kegg/compound/). Following annotation, the metabolites were subsequently mapped to the KEGG Pathway database (http://www.kegg.jp/kegg/pathway.html). The identification of pathways that are significantly enriched is accomplished by utilizing the P-value of a hypergeometric test for a specified set of metabolites.

**Statistical analysis**

All the data were expressed as mean ± standard error of mean (SEM). A t test or One-way ANOVA with Tukey post hoc test was used for the statistical evaluation of all parameters. A p value ≤0.05 was considered as statistically significant. Repeated measure one-way ANOVA was used to statistically analyze the body weight at various time points. All of the data were analyzed using the SPSS 21.0 software (SPSS Inc., Chicago, USA).

**Supplementary figure legends**

**Figure S1.** Behavioral assay of WT and *Csf1r^P853T/+^* mice. **(A)**Forced swimming test, **(B)** Sucrose preference test, and **(C)** Tail suspension test were used to analyze depressive behavior. **(D-F)** Attack test and **(G**) PPI test were to discover the schizophrenic behavior. n = 8 per group. The means SEMs are shown. **P < 0.01, ***P < 0.001.

**Figure S2.** Behavioral assay and histology of WT and *Csf1r^P853T/+^* mice at different age. **(A)**Morris Water Maze, and **(B)** Beam test were used to analyze cognitive and motor behavior. **(C)** LFB staining was used to discover the corpus callosum histology. n=8 per group. The means SEMs are shown. *P < 0.05, **P < 0.01, ***P < 0.001. The means SEMs are shown. *P < 0.05, ***P < 0.001. CSF-1R-KI stands for *Csf1r^P853T/+^*.

**Figure S3.** Pathology and imflammatory differences between WT and Csf1r^P853T/+^ mice. (A) Ultrastructural alteration under electron microscopy of *Csf1r^P853T/+^* mice. (B) Immunofluorence staining data of *Csf1r^P853T/+^* mice and WT mice. Red: Iba-1; Green: Csf1r; Orange: alpha-B; Blue: DAPI. (C) Inflammatory cytokines levels of *Csf1r^P853T/+^* mice.

**Figure S4.** Transcription and metabolic analysis of WT and Csf1r^P853T/+^ mice. (A) The bubble plot of *Csf1r^P853T/+^* most related gene pathways. (B) Heatmap reveals the differential metabolite levels between the WT and *Csf1r^P853T/+^* mice. The means SEMs are shown. *P < 0.05, ***P < 0.001.

**Figure S5.** Graphical summary.
